# Autophagy Induced by Areca Nut Extract Contributes to Decreasing Cisplatin Toxicity in Oral Squamous Cell Carcinoma Cells: Roles of Reactive Oxygen Species/AMPK Signaling

**DOI:** 10.3390/ijms18030524

**Published:** 2017-03-01

**Authors:** Zhi Xu, Chun-Ming Huang, Zhe Shao, Xiao-Ping Zhao, Meng Wang, Ting-Lin Yan, Xiao-Cheng Zhou, Er-Hui Jiang, Ke Liu, Zheng-Jun Shang

**Affiliations:** 1 The State Key Laboratory Breeding Base of Basic Science of Stomatology (Hubei-MOST) & Key Laboratory for Oral Biomedicine Ministry of Education, Wuhan University, Wuhan 430079, China; xuzhi_oms@whu.edu.cn (Z.X.); huangchunming89@126.com (C.-M.H.); shaozhe@whu.edu.cn (Z.S.); wangmeng29@hotmail.com (M.W.); yantinglin0929@hotmail.com (T.-L.Y.); 2016103040024@whu.edu.cn (X.-C.Z.); csujeh@163.com (E.-H.J.); 2 Department of Oral and Maxillofacial-Head and Neck Oncology, School and Hospital of Stomatology, Wuhan University, 237 Luoyu Road, Wuhan 430079, China; 3 Center of Stomatology, Tongji Hospital, Tongji Medical College, Huazhong University of Science and Technology, Wuhan 430000, China; topzxp2005@163.com

**Keywords:** areca nut extracts, reactive oxygen species, AMPK/mTOR signaling, autophagy, oral squamous cell carcinoma, cisplatin

## Abstract

Chewing areca nut is closely associated with oral squamous cell carcinoma (OSCC). The current study aimed to investigate potential associations between areca nut extract (ANE) and cisplatin toxicity in OSCC cells. OSCC cells (Cal-27 and Scc-9) viability and apoptosis were analyzed after treatment with ANE and/or cisplatin. The expressions of proteins associated with autophagy and the AMP-activated protein kinase (AMPK) signaling network were evaluated. We revealed that advanced OSCC patients with areca nut chewing habits presented higher LC3 expression and poorer prognosis. Reactive oxygen species (ROS)-mediated autophagy was induced after pro-longed treatment of ANE (six days, 3 μg). Cisplatin toxicity (IC_50_, 48 h) was decreased in OSCC cells after ANE treatment (six days, 3 μg). Cisplatin toxicity could be enhanced by reversed autophagy by pretreatment of 3-methyladenine (3-MA), *N*-acetyl-l-cysteine (NAC), or Compound C. Cleaved-Poly-(ADP-ribose) polymerase (cl-PARP) and cleaved-caspase 3 (cl-caspase 3) were downregulated in ANE-treated OSCC cells in the presence of cisplatin, which was also reversed by NAC and Compound C. Collectively, ANE could decrease cisplatin toxicity of OSCC by inducing autophagy, which involves the ROS and AMPK/mTOR signaling pathway.

## 1. Introduction

Oral squamous cell carcinoma (OSCC) is one of the most common kinds of malignancy in the head and neck region [1]. Patients with OSCC usually present with an advanced stage of the disease at their first diagnosis and treatment. Prognosis still remains unsatisfactory despite the advances in the multimodality treatment of advanced OSCC patients over the past few decades [2,3]. Consistent with the current guidelines, cisplatin-based chemotherapy remains the gold standard for advanced OSCC and could significantly improve the survival rate [4]. Cisplatin, which induces apoptotic pathways, was approved by the Food and Drug Administration (FDA) for the treatment of various kinds of solid carcinomas, such as head and neck carcinomas, including OSCC. Although cisplatin often leads to therapeutic effects, the development of chemo-resistance often causes chemotherapy failure with decreased toxicity to cancer cells, where there are various mechanisms [5]. Acquired chemoresistance will critically reduce the efficiency of chemotherapy drugs, including cisplatin. It was reported that the initial sensitivity of platinum-based chemotherapies in oral squamous cell was satisfied; however, over 70% of head and neck carcinoma patients may eventually suffer cancer relapse due to developed drug resistance [4].

Among numerous etiological factors, evident correlation has been noticed between areca nut chewing and the increased risk of OSCC, especially in the Indian subcontinent and Southeast Asia [6,7,8]. The areca nut is the seed of the palm plant *Areca catechu* L., which contains alkaloids including arecoline, arecaidine, guvacine, and guvacoline. It has been demonstrated that areca nut extract (ANE) and its containing alkaloids have genotoxic and cytotoxic effects and also have the potential for carcinogenesis [7,9,10]. However, its effects on the chemosensitivity of OSCC remains largely elusive.

Autophagy is an adaptive reaction to maintain energy homeostasis under various stresses such as hypoxia, starvation, ischemia/reperfusion, and so on, which can occur in both normal and cancer cells [11,12]. At present, autophagy has become a potential anticancer target both in cancer prevention and therapy, despite its controversial functions including OSCC [13,14,15,16,17]. Reactive oxygen species (ROS) can lead to various effects on different signaling pathways and results in genomic instability by inducing DNA damage. ROS induces autophagy, which in turn functions in reducing oxidative damage [18,19], so the ROS level could be associated with chemoresistance and cancer stem cells [20,21,22]. ANE is reported to induce the ROS in both cancer cells and normal oral epithelial cells [9,23]. It was also reported that ANE could induce autophagic flux through ROS [23]. Adenosine monophosate-activated protein kinase (AMPK) plays an important role in energy metabolism, which can also be triggered by oxidative stress [24]. AMPK activation is a well-known downregulator of mTOR activation, which is a key negative regulator to suppress autophagy. We then hypothesized that AMPK signaling pathway may be involved in autophagy induced by ANE. However, the underlying mechanism of correlations between the ROS/AMPK mediated autophagy and cisplatin resistance induced by ANE are not fully understood.

This study aims to investigate the effect of prolonged non-toxic ANE treatment on autophagy and cisplatin toxicity in OSCC cells. The roles of ROS/AMPK signaling pathways were revealed preliminarily in this process. Collectively, our results provide new insights into the correlation of areca nut usage with cisplatin toxicity in OSCC and are useful in finding novel strategies to optimize the current chemotherapeutic regimen of OSCC patients.

## 2. Results

### 2.1. Decreased Cisplatin Sensitivity and Higher LC3 Expression in OSCC Patients with Areca Nut Chewing

**Figure 1:**
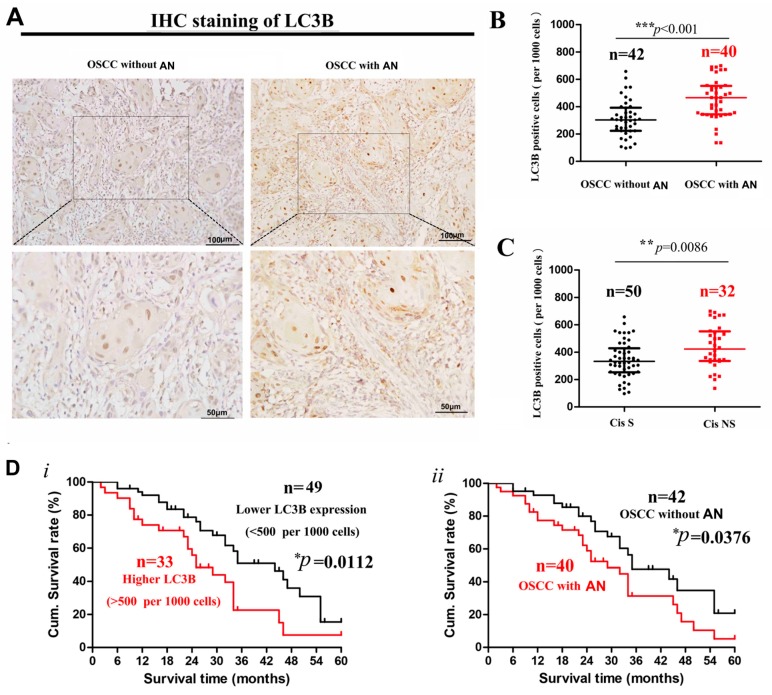
(**A**) Representative images of LC3B immunohistochemical (IHC) staining (×200 and ×400 magnification) in tumor sites of oral squamous cell carcinoma (OSCC) tissue samples with or without areca nut usage. (**B**) Box plots of the expression level of LC3B in tumor site comparing cisplatin sensitive vs. cisplatin non-sensitive group of advanced OSCC patients. *** *p*-value less than 0.001. (**C**) The expression levels of LC3B in the tumor specimens comparing OSCC with or without areca nut usage. ** *p* < 0.01. (**D**) Kaplan-Meier survival curves of overall survival rates were schemed in terms of LC3B expression and areca nut usage in OSCC patients, separately. Results were analyzed via log-rank test. Cis S: cisplatin sensitive group; Cis NS: cisplatin non-sensitive group; OSCC with AN: OSCC samples with areca nut chewing habit; OSCC without AN: OSCC samples without areca nut chewing habit.

A retrospective analysis of the advanced OSCC samples treated with cisplatin was performed in 82 advanced OSCC patients treated with cisplatin preoperatively. Our results revealed that samples with areca nut usage presented higher cisplatin resistance compared with the control (43.5% vs. 34.8%). Immunohistochemical (IHC) staining was conducted to evaluate the LC3 expression in tissue samples of the patients involved and showed that LC3 was expressed as puncta according to autophagosomes in cytoplasm (Figure 1A). LC3 expression was significantly higher in OSCC patients associated with areca nut chewing (Figure 1B). Meanwhile, the expression of LC3 was significantly higher in the cisplatin resistance group (Figure 1C). 

Survival curves were calculated for the 82 patients. Survival analysis was conducted to evaluate patient overall survival (OS) in terms of LC3 expression and areca nut chewing habit. The cumulative survival rates at 60 months was 18.5% and 10.8% in the OSCC patients with relatively higher and lower LC3 expression in tumor sites, respectively; this rate was 20.3% and 8.2% in those with and without areca nut usage, respectively. The differences in overall survival were both significant (Figure 1D). According to the results, it is speculated that ANE usage may be involved in cisplatin resistance and prognosis of OSCC patients, during which autophagy may play an important role.

### 2.2. Low-Dose Usage of Areca Nut Extract (ANE) Showed No Significant Effects on Cell Viability and Apoptosis of Oral Squamous Cell Carcinoma (OSCC) Cells

**Figure 2:**
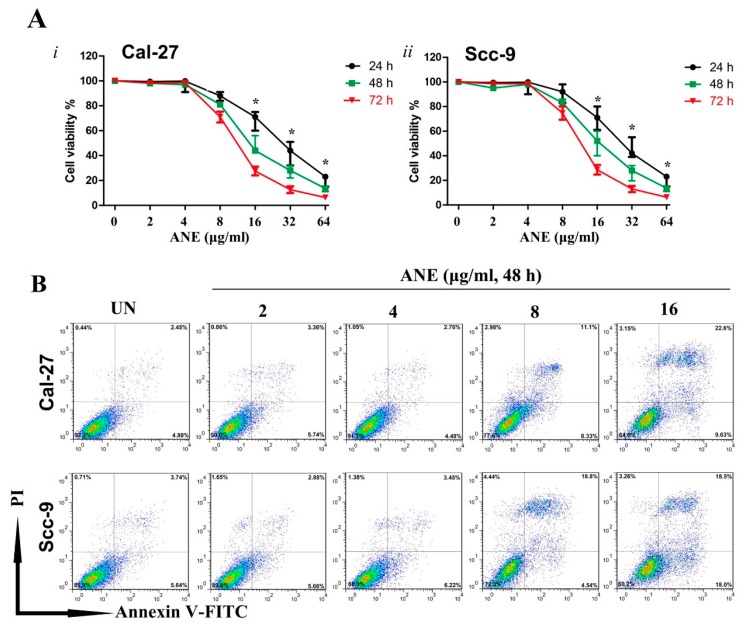

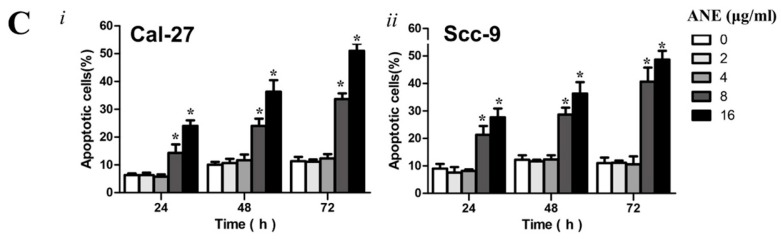
(**A**) Cell viability of Cal-27 and Scc-9 cells treated with various concentrations of ANE (0–16 μg/mL, 24, 48, and 72 h) analyzed by CCK-8 assay. (**B**) Cell apoptosis assay of Cal-27 and Scc-9 cells in the presence of various concentrations of ANE for 48 h presented as plots measured after Annexin-V and PI staining via flow cytometry. (**C**) Quantification for ANE-induced apoptosis cells analyzed by flow cytometry after Annexin V-PI staining. * *p* < 0.05 vs. the untreated group. Results are revealed as the mean ± SD of three independent experiments. UN: untreated with ANE.

To evaluate the effect of ANE on the OSCC cells, OSCC cell lines (Cal-27 and Scc-9) were applied for experiments in vitro. The CCK-8 assay suggested that ANE inhibits the cell viability in time- and dose-dependent manners within a dose range of 0–64 μg/mL. Cell viability was markedly decreased (up to 60%) in both OSCC cell lines with 32 μg/mL ANE for 24 h. The IC_50_ of ANE for Cal-27 and Scc-9 was 17 and 16.7 μg/mL in 72 h, respectively (Figure 2A, i and ii). Apoptosis analysis also revealed that apoptotic cells caused by ANE were significantly increased in dose- and time- dependent manners (Figure 2B,C). When treated with 1–4 μg/mL ANE, however, the viability of cells did not decrease significantly when compared with the control in varied time points (Figure 2A). Apoptosis analysis also showed no significant difference of apoptotic cells between ANE-treated and non-ANE-treated OSCC cells with low-dose ANE (1–4 μg/mL) (Figure 2B,C). To prevent the direct toxic effect caused by ANE, cells were treated with low-dose ANE, and non-toxic ANE (1 or 3 μg/mL) was used to treat Cal-27 and Scc-9, respectively, for 6 days. The surviving cells are designated for further research, and the OSCC cells of the negative control group were treated under the same conditions without ANE for same time during the next investigation.

### 2.3. Autophagy Induced by ANE Was Mediated by Reactive Oxygen Species (ROS)

**Figure 3:**
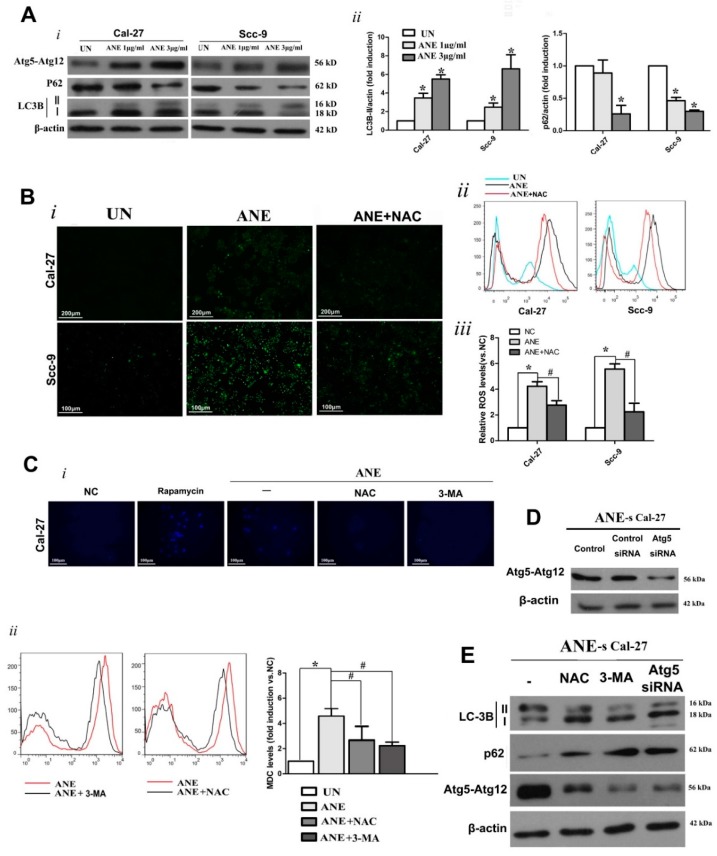
(**A**) Upregulated autophagy in Cal-27 and Scc-9 cells treated with ANE. OSCC cells were exposed to 1 or 3 μg/mL of ANE for 6 days. *i.* Effects of ANE on protein expression levels of Atg5–Atg12, p62, and LC3B in OSCC cells by Western blot. *ii.* Quantification for autophagy-essential proteins by Western blot analysis. * *p* < 0.05 vs. the untreated group (**B**) *i.* Detection of ROS generation labeled by 2′,7′-dichlorodihydrofluorescein diacetate (DCF-DA) via fluorescence microscopy in Cal-27 and Scc-9 cells under exposure of ANE (3 μg/mL) for 6 days, with or without NAC pretreatment. *ii.* Cellular ROS level detection with DCF-DA labeling by flow cytometry. *iii.* Quantification for ROS levels. Results are expressed as the mean ± SD of three independent experiments. * *p* < 0.05 vs. the untreated group, # *p* < 0.05 vs. the only ANE-treated group. (**C**) *i.* monodansylcadeverine (MDC) detection by fluorescence microscopy in Cal-27 cells treated with ANE and pretreatment with 3-MA or NAC, rapamycin pretreatment (1 μM, 3 h) were used for positive control. *ii.* Level detection and quantification of MDC in ANE-treated Cal-27 cells via flow cytometry with pretreatment with 3-MA or NAC. Results are expressed as the mean ± SD of three independent experiments. * *p* < 0.05 vs. the untreated group, # *p* < 0.05 vs. the only ANE-treated group. (**D**) Atg5–Atg12 expression was verified in Cal-27 and Scc-9 cells and analyzed by Western blot after Atg-5 siRNA knockdown. (**E**) Detection of indicated autophagy-essential proteins by Western blotting in ANE-treated Cal-27 cells with pretreatment of NAC, 3-MA, or siRNA-Atg5. Three independent experiments were conducted for Western blotting analysis.

**Figure 4:**
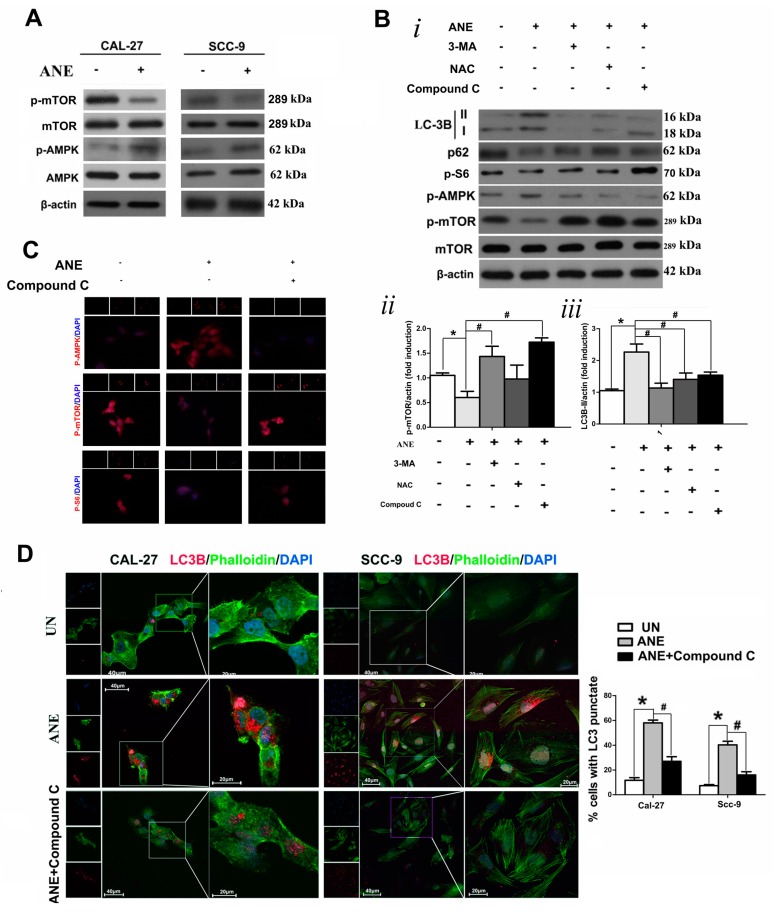

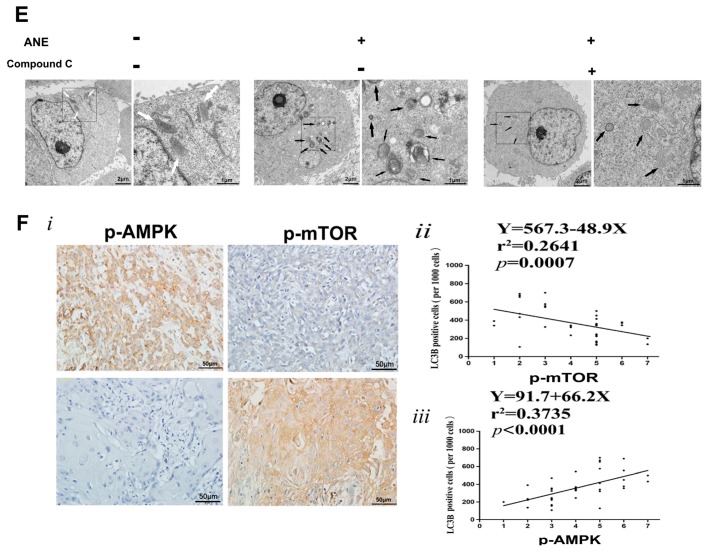
AMPK/mTOR signaling pathway involved in the ANE-induced autophagy process. (**A**) Western blot results of indicated protein expression (AMPK, p-AMPK, mTOR, and p-mTOR) in OSCC cells treated/untreated with ANE. (**B**) *i.* Western blot results of autophagy regulatory proteins and p-AMPK, p-S6, p-mTOR, and mTOR in Cal-27 cells: the cells were treated with ANE and/or pretreated with 3-MA, NAC, and Compound C, separately. *ii–iv.* Quantification for p-mTOR and LC3 via Western blot analysis. Three independent experiments were conducted, and results were expressed as mean ± SD. * *p* < 0.05 vs. the untreated group, # *p* < 0.05 vs. the only ANE-treated group. (**C**) Immunofluorescent staining of p-AMPK, p-mTOR, and p-S6 (red) in the presence of ANE combined with Compound C pretreatment. Nuclear counterstaining was performed with DAPI (blue). (**D**) Detection of LC3 immunostaining punctuate using confocal fluorescence microscope and*.* quantification for punctuate pattern of LC3 immunostaining. OSCC cells were exposed to 3 μg/mL of ANE for 6 days with or without NAC pretreatment. LC3 (red) expression was detected by immunostaining colocalized with the cytoskeleton revealed by phalloidin staining (green). Nuclear counterstaining was performed with DAPI (blue). Statistic results are revealed as the mean ± SD of three independent experiments. * *p* < 0.05 vs. the untreated group. # *p* < 0.05 vs. the only ANE-treated group. (**E**) Representative images of autophagosomes via TEM in ANE-treated Cal-27 cells. The cells were detected with pretreatment Compound C before ANE exposure or not. Normal mitochondria (white arrows) and ANE-mediated autophagosomes (black arrows) in Cal-27 cells via TEM were marked. (**F**) Correlations among p-mTOR or p-AMPK, with LC3 expression in tissue samples. *i*. Two representative IHC staining of p-mTOR and p-AMPK in advanced OSCC tissue specimens associated with areca nut chewing (×400 of magnification). *ii* and *iii*. Associations between LC3 and p-mTOR or p-AMPK expression levels via Spearman rank correlation analysis.

Higher LC3B-II expression levels in cells with ANE treatment groups were verified by Western blot (Figure 3A, i and ii). ANE resulted in a decrease in the autophagic flux marker SQSTM1/p62 (Figure 3A, i and ii), and the overexpression of Atg5–Atg12, which is essential for autophagosome formation (Figure 3A). Autophagy inhibitor 3-MA (2 mM) or siRNA-Atg5 (verified in Figure 4D) also led to an evident inhibition of ANE-induced autophagy (Figure 3C,E), while 1 μM rapamycin pretreatment (3 h) was used to detect autophagy as a positive control (Figure 3C). Intracellular ROS levels were measured using 2′,7′-dichlorodihydrofluorescein diacetate (DCF-DA) after treatment with ANE in both OSCC cell lines. A significant promotion of ROS level was evidently triggered in ANE-treated cells as marked by DCF-DA (Figure 3B). The ROS scavenger *N*-acetyl cysteine (NAC) was further applied to explore the protective effects against ANE-initiated ROS and autophagy. NAC (4 mM) was applied to pretreat OSCC cells for 3 h before ANE treatment. As expected, ROS levels were compromised after pretreatment with NAC (Figure 3B), whereas a higher level of monodansylcadeverine (MDC)-positive granular structures was present in ANE-treated cells. This means that NAC pretreatment could effectively reverse the ANE-induced elevation of MDC levels (Figure 3C). LC3B-II, Atg5–Atg12 overexpression, and SQSTM1/p62 downregulation caused by ANE could also be reversed by NAC pretreatment (Figure 3E). The above-mentioned results indicate that the increased ROS level is closely correlated with autophagy inducement under the exposure of ANE.

### 2.4. AMP-Activated Protein Kinase (AMPK)/mTOR Signaling Pathway Was Involved in ANE-Induced Autophagy

Western blot showed that AMPK phosphorylation was indicated as effectively overexpressed, and the expression of p-mTOR also decreased in both ANE-treated OSCC cell lines (Cal-27 and Scc-9) compared with the control (Figure 4A). To further determine whether the AMPK/mTOR pathway was involved in ANE-induced autophagy mediated by AMPK, we used the signaling inhibitor Compound C (50 μM) to pretreat Cal-27 cells for 2 h before each ANE exposure. Western blot results suggested that ANE-induced autophagy was partially reversed after Compound C pretreatment, indicating that the AMPK/mTOR pathway was involved in this autophagy process (Figure 4B). Furthermore, cell immunofluorescence staining demonstrated increased p-AMPK expression, whereas p-mTOR and p-S6 activation were downregulated in ANE-treated Cal-27 cells, which could be reversed by Compound C (Figure 4C). Additionally, formations of autophagosomes marked by LC3 using cellular immunofluorescence in OSCC cells also demonstrated that Compound C could decrease the LC3 level raised by ANE in OSCC cells in OSCC cells (Figure 4D). ANE-induced autophagosomes also significantly decreased in the presence of Compound C by transmission electron microscopy (TEM) scanning (Figure 4E). Meanwhile, the changes in AMPK signaling could be partially counteracted in the presence of NAC, further indicating that the ROS could at least be partially involved in signaling pathways (Figure 4B). 

In addition, the expression levels of p-AMPK and p-mTOR were measured by IHC staining in the specimens of 40 OSCC patients with areca nut chewing. IHC staining of both p-mTOR and p-AMPK was confined to the tumor cell membrane and cytoplasm, with minimal staining of the surrounding connective tissues (Figure 4F). The Spearman rank correlation coefficient test suggested that a markedly negative correlation was found between LC3 and p-mTOR (Figure 4F, ii), and that there was a significantly positive correlation between the LC3 and p-AMPK expression (Figure 4F, iii) in advanced OSCC patients with areca nut chewing. The above results indicated that the AMPK signaling pathway was involved in ANE-induced autophagy regulated by mTOR in OSCC.

### 2.5. ROS/AMPK Mediated Autophagy Decreased Cisplatin Sensitivity in OSCC Cells

**Figure 5:**
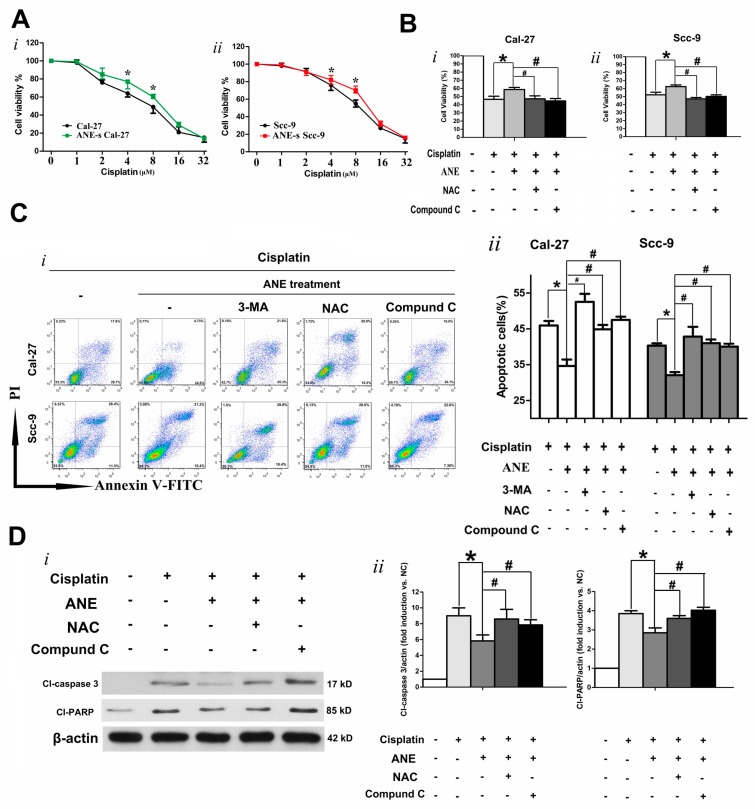
ANE-mediated autophagy associated with cisplatin resistance. (**A**) ANE downregulated cisplatin sensitivity in OSCC cell lines. After OSCC cells were treated with ANE (3 μg/mL) for 6 days, cisplatin (0–32 μM) was administrated for 48 h. *i* and *ii.* Cell viability was detected by CCK-8 assay in the presence of cisplatin for 48 h for Cal-27 and Scc-9 cells, separately. Results are revealed as the mean ± SD of three independent experiments. * *p* < 0.05 vs. the control group. UN: untreated with ANE as the control. (**B**) Cell viability of ANE (3 μg/mL, 6 days) treated Cal-27 and Scc-9 in the presence of cisplatin (IC_50_ concentration) for 48 h, with NAC or Compound C pretreatment or not. * *p* < 0.05 vs. non-ANE treatment group, # *p* < 0.05 vs. the only ANE-treated group. Three independent experiments were conducted and data was expressed as mean ± SD. (**C**) Cal-27 and Scc-9 cells were exposed to IC_50_ concentration of cisplatin for 48 h with or without pretreatment of ANE, 3-MA, NAC, or Compound C, separately. *i.* Cell apoptosis of OSCC cells induced by cisplatin presented as 2D density plots measured via flow cytometry after Annexin V-PI staining. *ii.* Quantification for apoptosis induced by cisplatin (IC_50_, 48 h) via flow cytometry after Annexin V-PI staining. Results are expressed as the mean ± SD of three independent experiments. * *p* < 0.05 vs. non-ANE-treated groups, # *p* < 0.05 only ANE-treated group in the presence of cisplatin. (**D**) *i.* Western blot results of cl-caspase 3 and cl-PARP protein expressions in Cal-27 cells in the presence of cisplatin under various condition. *ii.* Quantification for apoptosis-related proteins induced by cisplatin (IC_50_, 48 h) via Western blot analysis. Three independent experiments were conducted. * *p* < 0.05 vs. non-ANE-treated groups, # *p* < 0.05 only ANE-treated group in the presence of cisplatin.

OSCC cells were exposed to cisplatin (0–32 µM) for 48 h after treatment with 3 μg/mL ANE for 6 days. The CCK-8 assay demonstrated that the ANE-treated cell group showed elevated cell viability in comparison with the control group (Figure 5A). The IC_50_ of control Cal-27 and Scc-9 were approximately 7.85 and 11.8 µM, whereas the IC_50_ of ANE-treated Cal-27 and Scc-9 were 10.25 and 14.3 µM for 48 h, respectively. When the OSCC cells were exposed to the IC_50_ concentration of cisplatin for 48 h, cell viability of OSCC was assessed using the CCK-8 assay, results showed that OSCC cell viability was increased with ANE pre-treatment in the presence of cisplatin (Figure 5B). Additionally, the apoptotic cells induced by cisplatin in OSCC cells were also detected by double staining with Annexin V and PI before analysis using a FACS flow cytometer. Results of the double staining with Annexin V-PI showed that cisplatin-induced apoptotic cells were downregulated in ANE-treated OSCC cells (Figure 5C). It was revealed that survived OSCC cells were increased in cells with ANE treatment in the presence of cisplatin for 48 h with IC_50_ concentration (Figure 5B,C). These results indicated that ANE treatment could decrease cisplatin toxicity in OSCC cells. However, cisplatin toxicity could be significantly enhanced in ANE-treatment OSCC cells as showed by cell viability, by blocking autophagy during ANE treatment using 3-MA, NAC, and Compound C (Figure 5B). By blocking autophagy with pretreatment using 3-MA or NAC during ANE treatment, the cisplatin-induced apoptosis ratio was enhanced in ANE-treated OSCC cells (Figure 5C). Compound C could also significantly reverse decreased toxicity of cisplatin in ANE-treated Cal-27 cells (Figure 5C). Furthermore, after cisplatin treatment for 48 h in Cal-27 cells, Western blot showed that cl-PARP and cl-caspase 3 were downregulated in ANE-treated Cal-27 cells compared with the control cells; the downregulated expression could be partially reversed by NAC and Compound C, separately (Figure 5D). These results demonstrate that ANE-induced ROS/AMPK-mediated-autophagy is related to decreased cisplatin toxicity and that the reversion of autophagy by NAC/Compound C could enhance cisplatin sensitivity.

**Figure 6:**
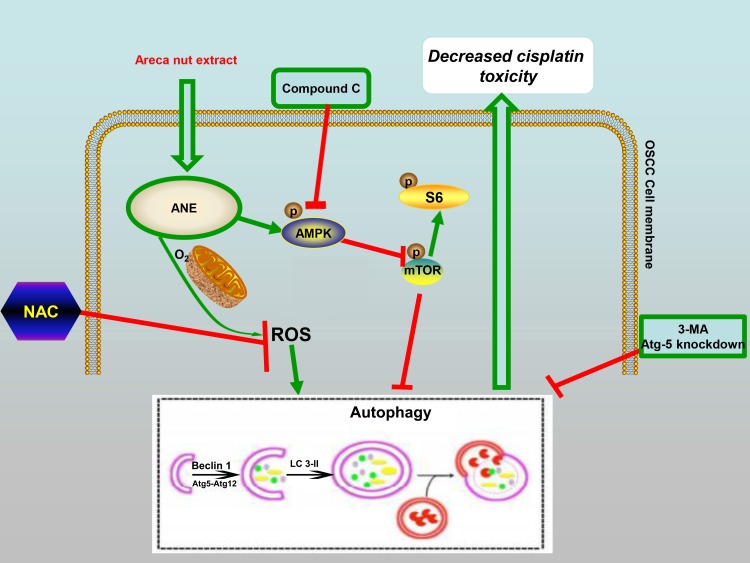
Schematic summarizing how ANE decreases cisplatin toxicity in OSCC cells by inducing autophagy, and that the ROS and AMPK/mTOR pathways were involved in this process. Non-toxic concentration of ANE could induce autophagy via activation of the AMPK/ROS signaling pathway coincided with the activation of ROS accumulation. The ANE-induced autophagy was related with decreased cisplatin toxicity in OSCC cells.

Taken together, our results indicate that ANE could decrease the cisplatin toxicity of OSCC cells by inducing autophagy and that the ROS and AMPK/mTOR pathways are involved in this process (Figure 6).

## 3. Discussion

Areca nut chewing is closely associated with oral cancers by promoting oral submucous fibrosis (OSF) formation [25]. The relationships between OSF-associated OSCC and drug resistance have not been well addressed in the literature. Our present study revealed that the overall survival rate of OSCC patients in advanced clinical stages, who were treated with chemotherapy based on cisplatin, was associated with areca nut usage. In addition, areca nut chewing OSCC patients showed a higher rate of cisplatin resistance and upregulated LC3 expression in tissue samples, indicating that ANE usage could contribute to the resistance of cisplatin in which autophagy may be involved. Consistent with a previous study [26], the higher expression of LC3 was associated with poorer prognosis according to our results. To our knowledge, we were the first to identify the differences in OSCC patients with or without ANE usage in terms of cisplatin resistance, autophagy, and prognosis, though these results are preliminary and have limitations. An attempt was made in the present study to evaluate the prognosis of locally advanced OSCC in terms of LC3 expression and areca nut usage, separately. However, prognosis of OSCC can be affected by many factors, such as clinic stages and different clinical strategies, including surgery techniques and radiotherapy/chemotherapy methods. While the subjects recruited were all advanced OSCC treated with cisplatin preoperatively from one hospital, it can be reasonably speculated that several biases still exist since the prognosis of OSCC was evaluated solely on LC3 expression or areca nut usage when this retrospective analysis was conducted. We did not address the above issues comprehensively, which is one of the limitations of this study. To gain further understanding of the clinical outcomes for OSCC patients in terms of areca nut usage, studies comparing different clinicopathological features and clinical treatment strategies, particularly prospective cohort studies, are still necessary.

Autophagy is recognized as being closely related with the prognosis of patients with carcinomas, including OSCC [14,16,26]. The functions of autophagy in OSCC remain controversial. Autophagy can inhibit OSCC cells from progression and could promote cell death by mediating apoptosis [17]. Autophagy could also possess anti-apoptotic properties to induce the formation of drug-resistant cancer cells [14,15]. Autophagy is believed to perform a “double-edged sword” function in the progression and treatment of cancer [27,28]. Although ANE-induced autophagy has been observed in oral cancer cells, the underlying mechanisms still require further investigation. Furthermore, studies involving different conditions in different cells could provide additional information in the identification of ANE-induced autophagy. Given that ANE could induce both apoptosis and autophagy, whether ANE is a factor to improve resistance for antitumor treatment requires further research. In this present study, we used a non-toxic dose to prevent direct toxic effect by ANE. ROS levels were also improved in cells treated with a prolonged non-toxic ANE, and the relationship between ROS and autophagy was demonstrated. ROS reportedly functions in cancer drug resistance [22]. ANE can induce the generation of ROS in both normal and cancerous epithelium cells [23,29,30]. ROS is also associated with autophagy. Notably, autophagy could act as a primarily survival mechanism in response to ROS, and the removal of damaged mitochondria and oxidized proteins by autophagy helps cells survive [18]. As a kind of ROS inhibitor, NAC could promote cisplatin sensitivity in cells treated with ANE. A high level of ROS is reportedly associated with chemoresistance, and NAC could counteract the effect by decreasing the ROS level. Our study demonstrated that NAC inhibited ANE-induced autophagy and enhanced the apoptotic rate when ANE-treated OSCC cells were in the presence of cisplatin. However, further mechanisms are still needed to demonstrate the function of ROS in the chemoresistance of OSCC. Interestingly, arecoline, a major alkaloid in ANE, has been demonstrated to induce ROS and cell death but not autophagy [29,30]. To identify the specific compounds of ANE that lead to autophagy, a previous study has revealed that ANE at 100–300 K, which contains several kinds of inorganics, performs an important function in the autophagy process [23]. It should be mentioned that, in this study, we did not isolate any active ingredients from the ANE to investigate their functions separately, and only approached its human consumption as a chewing habit. Furthermore, cells were treated using a non-toxic dose for a prolonged period of time (6 days) instead of a shorter time treatment (less than 72 h) with a higher/toxic dose directly. We suppose that this model could better mimic the habit of chewing areca nut in vitro, since areca nuts are rarely used in toxic doses of short duration as a personal habit.

Our results also emphasized common mechanisms, namely the AMPK/mTOR pathway, which regulated ANE-mediated autophagy in the present study. AMPK is usually activated in response to a variety of extracellular and intracellular stresses, including oxidative stress-induced autophagy [31,32]. In particular, AMPK performs a critical function in cell growth, cell metabolism, apoptosis, and autophagy in mammalian cells [31,32,33,34,35,36]. P70S6K, a downstream of mTOR Complex 1, is involved in many biological functions through mTOR activation [37]. We demonstrated that AMPK activation combined with the inactivation of mTOR and P70S6k was induced by ANE in OSCC cells in the current study. Compound C was also introduced to determine whether mTOR signaling was involved in this process by evaluating the phosphorylations of downstream effectors including p70S6 kinase. Furthermore, p-mTOR and p-P70S6k mediated by the administration of ANE in OSCC cells could be reversed by pre-treatment with Compound C, suggesting that mTOR signaling is a mediated link between AMPK signaling and autophagy process with ANE treatment. Pathway inhibition by Compound C could partially counteract ANE-induced autophagy, as revealed by the ratio of LC3-II/β-actin and the accumulation of autophagic vacuoles by TEM scanning. The expression levels of p-AMPK and p-mTOR were also significantly associated with LC3 expression in areca nut-associated OSCC specimens. Thus, we revealed that autophagy induced by ANE was not only medicated by the ROS pathway but also the AMPK/mTOR pathway. It is another investigation that the autophagy mediated by ANE is dependent on, at least partially, the AMPK/mTOR pathway. ROS could stimulate the activity of AMPK based on the previous study in [38]. It was also indicated that ROS was involved in the AMPK pathway as, according to our results, the NAC could partially reverse the abnormal expressions of p-AMPK in ANE-treated Cal-27 cells. However, as we did not focus on the potential associations between the ROS and AMPK/mTOR pathway in the current study, corresponding investigations are still required. Furthermore, it has been reported that arecoline, as a major alkaloid of the areca nut, could inhibit AMPK through the ROS to induce apoptosis, while the 100–300 K ANE could increase the phosphorylation of AMPK [39]. It can be speculated that different mechanisms regulated by the areca nut extract exist and that further studies are still required.

Cisplatin-based chemotherapy is recommended for the treatment of advanced-stage cancers, including OSCC. However, cancer cells could become resistant to drugs, thus limiting the effects of chemotherapy [5]. Our results reveal a regulatory model of the ANE that affects cisplatin sensitivity via inducing the ROS and AMPK signaling pathways, demonstrating downregulated cisplatin toxicity through elevated autophagy activity. Cell viability assay and apoptosis analysis indicated that ANE-treated OSCC cells show higher cell viability and a lower apoptotic ratio in the presence of cisplatin, whereas 3-MA, NAC, and Compound C may reverse cisplatin resistance. According to our results revealed by Western blot, downregulation of cl-PARP, and cl-caspase 3 could be caused by the ANE administration in Cal-27 cells in the presence of cisplatin. It can be reasonably speculated that the expression of cl-PARP and cl-caspase 3 may be related with decreased toxicity in the presence of cisplatin, in which ROS/AMPK-mediated autophagy by ANE could participate. However, more specified mechanisms were not revealed in the present study and require further research.

## 4. Materials and Methods

### 4.1. Patients and Tissue Specimens

Locally advanced OSCC patients were involved from the Department of Oral and Maxillofacial–Head and Neck Oncology, School and Hospital of Stomatology, Wuhan University, Wuhan, China (*n* = 82). There were 40 patients with an areca nut chewing habit and the other 42 patients had never chewed areca nut. All patients were treated with preoperative chemotherapy based on cisplatin. According to the ‘Response Evaluation Criteria in Solid Tumors’ of the World Health Organization, OSCC patients with stable or progressed outcomes were included in the cisplatin-resistant OSCC group, whereas patients who showed a partial or complete response of cisplatin were classified as the cisplatin-sensitive group. Tissue specimens of each patient were used for further analysis after surgery for research usage only. Hematoxylin–eosin (HE) stained tissue sections of all samples were again assessed to confirm the correct diagnosis of OSCC. A routine recall schedule combined with periodical phone calls was applied to obtain follow-up data. This retrospective study was approved by the institutional ethical review boards of the hospital, and informed consent was obtained from all patients. The study was approved by the Medical Ethics Committee of School and Hospital of Stomatology, Wuhan University (Approval number: 2013LUNSHENZI103, 1 March 2013).

### 4.2. Chemicals and Reagents

Monodansylcadaverine (MDC); 3-methyladenine (3-MA); rapamycin; *N*-acetyl-l-cysteine (NAC); Compound C; 2′,7′-dichlorodihydrofluorescein diacetate (DCF-DA); and cisplatin were obtained from Sigma-Aldrich (St. Louis, MO, USA). LC3B and Atg5 antibodies were also purchased from Sigma-Aldrich. Control siRNA and Atg5-siRNA were obtained from Life Technologies (Carlsbad, CA, USA). Cl-caspase 3; mammalian target of rapamycin (mTOR); p-mTOR (Ser2448); AMPK; phospho-AMPK (Thr172); p70S6kinase (S6); and phosphor-S6 (Thr389) antibodies were purchased from Cell Signaling Technology (Danvers, MA, USA). Antibodies against SQSTM1/p62 and cl-PARP were obtained from Epitomics (Burlingame, CA, USA); and β-actin and CCK-8 were purchased from Beyotime Biotechnology (Shanghai, China). The FITC Annexin–V Apoptosis Detection Kit I was purchased from BD Biosciences (San Jose, CA, USA).

### 4.3. Areca Nut Extract (ANE) Preparation

The ANE preparation and fractionation were conducted according to a previous description in [23,29,30]. In brief, 30 g of tender areca nut from dried nuts were ground and extracted using de-ionized water for 1 h at 4 °C. Next, the squeezed extract was centrifuged at 12,000× *g* for 10 min. The supernatant was harvested, filtered, and concentrated for lyophilization, before the weighed dry powder was dissolved in deionized water as ANE for research use and stored at −20 °C.

### 4.4. Cell Lines and Culture

OSCC cell lines Cal-27 and Scc-9 were purchased from the American Type Culture Collection (ATCC, Manassas, VA, USA). Cal-27 cells were cultivated in DMEM (Hyclone, South Logan, UT, USA) and added to 10% fatal bovine serum (FBS) (Gibco, Grand Island, NY, USA). Scc-9 cells were cultivated in DMEM-F12 (Hyclone) supplemented with 10% FBS (Gibco) added to 400 ng/mL hydrocortisone. Both cell lines were cultured at 37 °C in 5% CO_2_, and the medium was changed every two to three days routinely upon reaching 70%–80% confluence of cells. The cells were plated at 30%–40% confluence in a medium supplemented one day before treatments. 

### 4.5. Cell Viability Assay

Cell viability was detected by the Cell Counting Kit-8 (CCK-8) assay, which was conducted according to the manufacturer’s instructions. Briefly, OSCC cells (Cal-27 and Scc-9) were seeded in 96-well culture plates with the different treatments as indicated. The ANE or cisplatin was added to the cell culture at varying concentrations after cell seeding, and cell proliferation was determined after incubation for 24, 48, and 72 h. The cells were then incubated with 10 μL of CCK-8 and 100 μL of DMEM at 37 °C for another 2 h, after which the absorbance at 450 nm was measured with a micro-plate reader (Thermo MutliscanMK3; Thermo Fisher Scientific, Waltham, MA, USA). Cell viability curves were generated using a colorimetric assay.

### 4.6. Intracellular Reactive Oxygen Species (ROS) Level Detection

The redox-sensitive fluorescent dye DCF-DA was used for labeling the intracellular ROS level. DCF-DA was used for the ROS as described previously. The approximately treated OSCC cells were washed with phosphate buffer saline (PBS) and incubated with 10 μM DCF-DA for 30 min subsequently. After the removal of DCF-DA and two washes with PBS, the cells were collected with the PBS. Cellular ROS levels were detected and determined by a fluorescence microscope (Leica Microsystems, Wetzlar, Germany) and FACS flow cytometer (BD Biosciences), and the data analyzed using FlowJo software (Version 7.6, Tree Star, Ashland, OR, USA). ROS levels were displayed as fold changes compared with the control.

### 4.7. Cellular Immunofluorescence of OSCC

Immunofluorescence staining was conducted in fixed cells with various treatments as indicated. In brief, the cells were seeded on sterile glass cover slips at a density of 40,000 cells per cover. After incubation according to the research design, the cells were fixed with 4% paraformaldehyde for 15 min, washed three times with PBS, treated with 0.1% Triton X-100 for 5 min, blocked with 10% non-immune goat serum for 1 h at room temperature, and then incubated with antibodies (LC3B, p-AMPK, p-mTOR and p-S6) in 0.1% Triton X-100 at 4 °C overnight. After being washed twice, the cells were incubated with Cy3-conjugated goat anti-rabbit IgG (1:200) for 1 h at 37 °C. Cellular cytoskeleton and nuclei were labeled by 20 μg/mL FITC-conjugated phalloidin for 40 min, and 0.1 μg/mL 4,6-diamidino-2-phenylindole (DAPI) for 10 min at 37 °C, respectively. The coverslips were then mounted on a microscope slide with the embedding medium. Afterward, cells were examined and photographed using traditional or confocal fluorescent microscopy (Leica, Wetzlar, Germany).

### 4.8. Monodansylcadaverine (MDC) Staining

Cellular MDC detection was used to label acidic vesicular organelle in the autophagy process. Briefly, OSCC cells were treated accordingly after cell seeding at a density of 40,000 cells per cover on sterile coverslips overnight. The cells were incubated with 0.05 mM MDC at 37 °C for 30 min in the dark. Cells were washed three times with PBS and immediately analyzed under a Leica fluorescence microscope (Leica, Germany). The stained cells were then analyzed using a FACS flow cytometer (BD Biosciences).

### 4.9. Transmission Electron Microscopy (TEM) Analysis

TEM analysis was conducted to examine the autophagosome. The cells were approximately treated and harvested, fixed in 2.5% glutaraldehyde overnight at 4 °C, and postfixed in 1% osmium tetroxide with 0.1% potassium ferricyanide. After dehydration using a graded series of ethanol (30%–90%), the samples were embedded in Spurr’s epoxy resin and cut into ultrathin sections (80 nm). These ultrathin sections were then stained with 2% uranyl acetate and observed using a transmission electron microscope (Hitachi H-600, Hitachi, Tokyo, Japan).

### 4.10. Flow Cytometry Analysis of Cell Apoptosis

Following the manufacturer’s protocol (BD Bioscience), the apoptosis assay was conducted by flow cytometry using the Annexin V-FITC Apoptosis detection kit. The OSCC cells were seeded in six-well plates at 5 × 10^5^ cells/well, followed by various treatments. After incubation, the cells were collected, washed twice with PBS, suspended with 100 μL of 1× binding buffer, and stained with 5 μL of Annexin V and/or 5 μL of propidium iodide (PI) for 15 min in the dark according to the manufacturer’s instructions. After adding another 400 μL of 1× binding buffer, the stained cells were then analyzed using a FACS flow cytometer (BD Biosciences). Data were analyzed using FlowJo software (Version 7.6, Tree Star, Ashland, OR, USA).

### 4.11. Small Interfering RNA (siRNA) Knockdown

In short, OSCC cells were transfected with ATG5 siRNA (ID: 4392420, Ambion/Life Technologies, Carlsbad, CA, USA) using Lipofectamine 2000 (No. 11668019, Life Technologies) according to the manufacturer’s instructions. After a 48 h culture, knockdown efficiency was assessed at the protein level by Western blot analysis. Nonspecific siRNA (Negative Control-1 siRNA; AM4636, Life Technologies) was used as a negative control consistent with the manufacturer’s protocol. After 48 h treatment, Atg5 knockdown cells were confirmed by Western blot analysis and then subjected to further analysis. 

### 4.12. Western Blot Analysis

Total proteins from treated OSCC cellular lysates or concentrated conditioned media from cell cultures were prepared as previously described [40]. After quantifying protein concentration using the bicin-choninic acid (BCA) method, an equal amount of protein (80 μg) was resolved on 8%–12% sodium dodecyl sulfate-polyacrylamide gels electrophoresis (SDS–PAGE), and electrophoretically transferred to polyvinylidene fluoride (PVDF) membranes in the SDS-electroblot buffer (25 mM Tris-Cl, 192 mM glycine, 20% methanol, pH 8.3). Antibodies including LC3B, p62, Atg5-Atg12, Cl-PARP, Cl-caspase 3, phosphor-AMPK, AMPK, phosphor-S6, S6, p-mTOR, mTOR, and β-actin were used in the Western blot of the present study. The membranes were cultured at 4 °C overnight with the corresponding primary antibodies in blocking solution, washed three times with TBST at room temperature for 10 min, and incubated with secondary antibodies for 1 h at room temperature. Bound secondary antibodies were visualized by incubation with enhanced chemiluminescence (ECL) for 3 min after three washes for 10 min in TBST. The membranes were then stripped for reprobing at room temperature for 15 min using a mild antibody stripping buffer. Finally, the membranes were washed twice in TBST at room temperature for 10 min. The bound secondary antibody was visualized by ECL. Quantification and analysis of Western blot results were performed using Quantity One software (Bio-Rad, Hercules, CA, USA).

### 4.13. Immunohistochemistry (IHC) Staining and Evaluation

IHC staining was performed to detect the protein expression of LC3, p-AMPK, and p-mTOR in the tissue specimens. Tumor tissues were fixed in formalin, embedded in paraffin, sectioned to 4 μm, and mounted on slides. Immunohistochemical staining was performed to detect the protein expression of transplanted tumor sections. After deparaffinization and rehydration, antigens on the sections were retrieved by boiling in 10 mM sodium citrate buffer (pH 6.0) for 10 min. The activity of endogenous peroxidases was quenched by incubation in 3% H_2_O_2_ for 20 min at room temperature. After three washes with PBS, the slides were blocked for 30 min with 100 µL of normal goat serum for 1 h at room temperature. The slides were then incubated with primary antibodies (LC3B, p-AMPK, and p-mTOR) at 4 °C overnight and second antibodies for 1 h at room temperature. Stained slides were visualized using diaminobenzidine as a chromogenic substrate and counterstained with hematoxylin. Negative controls (without primary antibody incubation) were used in each immunostaining step. Immunohistochemistry evaluation was performed according to previously published protocols [26,41]. Briefly, a total of 5 × 200 tumor cells were counted to calculate the LC3B positive cells in each section. Staining of p-AMPK and p-mTOR was assessed using a score system (0–7) according to previous research in [41]. In short, a minimum of five different fields was analyzed in each section. Staining intensity was scored as follows (magnification ×40): 0, absence of staining; 1, weak staining; 2, moderate staining; and 3, intense staining. The proportion of staining (magnification ×200) was evaluated as follows: 0, no staining of cells in microscopic field; 1, <25% of cells positive; 2, 25%–50% of cells positive; 3, 50%–75% of cells positive; and 4, >75% of cells positive. By adding both scores together, the final immunostaining score of each specimen was finally defined as an average score (0–7) of fields observed.

### 4.14. Statistical Analysis

Statistical analysis was performed using GraphPad Prism (Version 5.01, La Jolla, CA, USA) statistical packages. Each experiment was repeated for at least three times to control bias. Values were presented as medians and interquartile ranges for LC3 comparison in human tissue samples. For other experimental data, results were expressed as mean values ± standard deviation (SD). The Student’s *t*-test, Mann–Whitney U-test, and one-way ANOVA followed by Student–Newman–Keul tests were conducted accordingly. A Spearman rank correlation analysis was performed to analyze the correlations between variables. A Kaplan–Meier survival curve and log-rank test were applied for the survival analysis of patients. A *p*-value of less than 0.05 was considered statistically significant.

## 5. Conclusions

In summary, the present study indicated that the ANE could induce autophagy in OSCC cells by prolonged treatment with a non-toxic dose. The ANE increased intracellular ROS, which could be counteracted by NAC. The phospho-AMPK level increased while the phospho-mTOR and phospho-S6 levels decreased in the presence of the ANE, which could be counteracted by Compound C. Inhibition of autophagy by 3-MA, NAC, and Compound C could attenuate this resistance to promote cell deaths in the presence of cisplatin (Figure 6). Accordingly, our results indicated that OSCC patients with areca nut chewing habits showed less chemosensitivity of cisplatin with a relatively higher level of LC3B expression. Collectively, ROS/AMPK-mediated autophagy could be proposed as a promising therapeutic target for increasing chemosensitivity in advanced OSCC with areca nut usage.

## Abbreviations

OSCC	oral squamous cell carcinoma
ANE	areca nut extract
ROS	reactive oxygen species
CCK-8	cell counting kit-8
MDC	monodansylcadeverine
NAC	*N*-acetyl cysteine
DCF-DA	2′,7′-dichlorodihydrofluorescein diacetate
3-MA	3-methyladenine
LC3	microtubule-associated protein 1 light chain 3
Atg5	autophagy related 5
Atg12	autophagy related 12
SQSTM1	sequestosome 1
DAPI	4′,6-diamidino-2-phenylindole
IC50	50% inhibitory concentration
IHC	immunohistochemical
Cl-PARP	cleaved-Poly-(ADP-ribose) polymerase
AMPK	adenosine monophosate-activated protein kinase
mTOR	mechanistic target of rapamycin
DMEM	Dulbecco’s modified Eagle’s medium
TEM	transmission electron microscopy
